# The association between perirenal fat volume and nephrolithiasis: A Bloemfontein study

**DOI:** 10.4102/jcmsa.v4i1.319

**Published:** 2026-05-30

**Authors:** Jozanne Voigt, Eugene Loggenberg, Mari Wentzel

**Affiliations:** 1 Department of Clinical Imaging Sciences, Faculty of Health Sciences, University of the Free State, Bloemfontein, South Africa

**Keywords:** unilateral nephrolithiasis, perirenal fat volume, fat distribution storing pattern, perirenal fat thicknes, gender

## Abstract

**Background:**

Nephrolithiasis is a well-recognised global health burden associated with significant morbidity. Recent studies have demonstrated a potential association between perirenal fat volume (PFV) and nephrolithiasis, particularly among males, likely related to sex-related differences in visceral fat distribution. However, this association has not yet been investigated within the South African population. We explored the association between PFV and nephrolithiasis by comparing the PFV surrounding a stone-bearing kidney with the PFV surrounding a non-stone-bearing kidney in the same individual in both male and female patients.

**Methods:**

A retrospective cross-sectional study was conducted at Universitas Academic Hospital in South Africa on patients diagnosed with unilateral nephrolithiasis. Manual tracing software was applied to non-contrasted computed tomography abdomen studies to calculate the PFV. The PFV difference surrounding normal and pathological kidneys was calculated and described using the Signed-Rank test for paired data.

**Results:**

A total of 140 patients were included, of which 91 were males and 49 were females. The PFV surrounding the stone-bearing kidney was statistically significantly higher than the PFV surrounding the non-stone-bearing kidney (*p* < 0.0001).

**Conclusion:**

A significant association between PFV and nephrolithiasis was found.

**Contribution:**

This study provides the first South African evidence of a significant association between perirenal fat volume and unilateral nephrolithiasis. It supports the potential use of perirenal fat thickness as a simpler radiological marker for future nephrolithiasis risk assessment and research in South African clinical practice.

## Introduction

Nephrolithiasis is a widely recognised condition and a substantial global health burden, impacting individuals across all demographics.^1,2^ Studies suggest a higher prevalence in men compared to women.^1,2,3^ Controversy and debate exist as to why there is an association between nephrolithiasis and the male or female sex.

Risk factors of nephrolithiasis are well known and studied. Risk factors include, but are not limited to dehydration, elevated body mass index (BMI), dietary habits, urinary tract infections and medications.^2,3,4^ Obesity or a BMI > 30 kg/m^2^ contributes to urine composition changes, which predispose individuals to stone formation.^4,5,6,7,8^ Body mass index, however, can unfortunately not distinguish muscle from fat contents and thus adipose tissue itself must be studied rather than BMI as a whole.^4,9^ Adipose tissue functions as a metabolically and hormonally active endocrine organ and can be divided into subcutaneous and visceral adipose tissue.^4,9,10,11^

Visceral perirenal adipose tissue, or perirenal fat volume (PFV), has recently been thought to be associated with nephrolithiasis.^4,10^ Perirenal fat volume represents the fat volume surrounding the kidney, anatomically enclosed within the renal fascia.^10,11^ When comparing the PFV around a healthy kidney to that around a kidney with a stone, three recent studies found that the PFV was significantly larger around the kidney with the stone than around the non-stone-bearing kidney.^4,10^

Recent years have provided evolution with regard to the radiological field. Perirenal fat volume can be calculated by applying 3D volumetrics or manual tracing software to ultrasound (US), computed tomography (CT) or magnetic resonance imaging (MRI).^12^

If the fat volume surrounding the kidney can be measured, a possible causative link between PFV and nephrolithiasis can be proposed.

Two recent studies suggested an association between perirenal fat thickness (PFT) and PFV^9,13^ simply by reasoning that the higher the amount of fat surrounding the kidney, the larger the distance between the posterior border of the kidney and the posterior abdominal wall would be.

To contribute to the global understanding of the pathophysiology and risk factors of nephrolithiasis, we aimed to investigate the radiological association between PFV, PFT, nephrolithiasis, and biological sex within a South African population.

## Research methods and design

Non-contrast abdominal CT scans were obtained with a slice thickness of 1.25 mm using a Discovery HD 750, 64-slice scanner. Manual tracing software was employed to calculate the variables within the perirenal fascia on these images. Measurements were made by the same radiology registrar in order to calculate average values for PFV and PFT.

**FIGURE 1:**
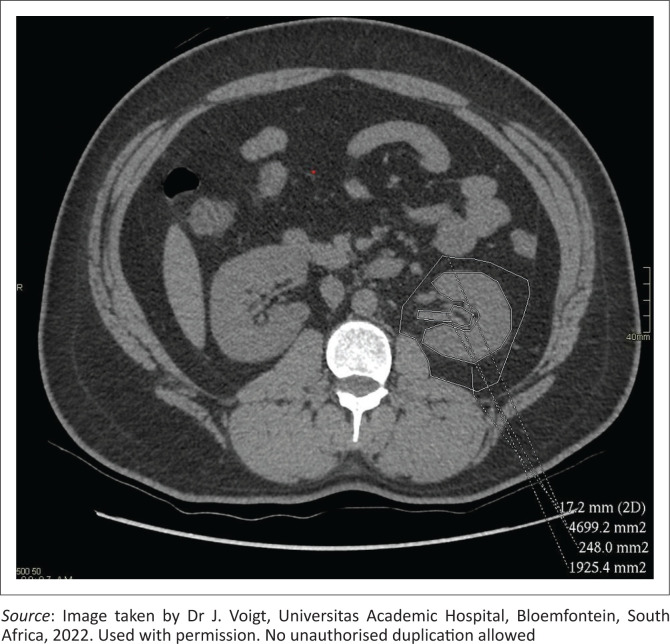
Calculating perirenal fat volume and perirenal fat thickness with manual tracing software at the level of the renal vein on non-contrasted axial computed tomography.

For each patient, biological sex and lateralisation of the stone were documented. On the axial view, at the level of the renal vein, the kidney area, hilum area and perirenal fat area bordered anteriorly by Gerota’s fascia and posteriorly by Zuckerkandl’s fascia were calculated for both pathological and healthy kidneys (see Figure 1 – example image obtained from one of the study participants).^10^ The borders of Gerota’s and Zuckerkandl’s fascia can be visualised as thin, soft tissue density lines within the retroperitoneum, surrounding the perirenal fat, the kidney and the renal hilum. The perirenal fat can easily be differentiated from the kidney, renal hilum and fascial lines by its low negative Hounsfield unit compared to higher positive soft tissue density.

**FIGURE 2:**
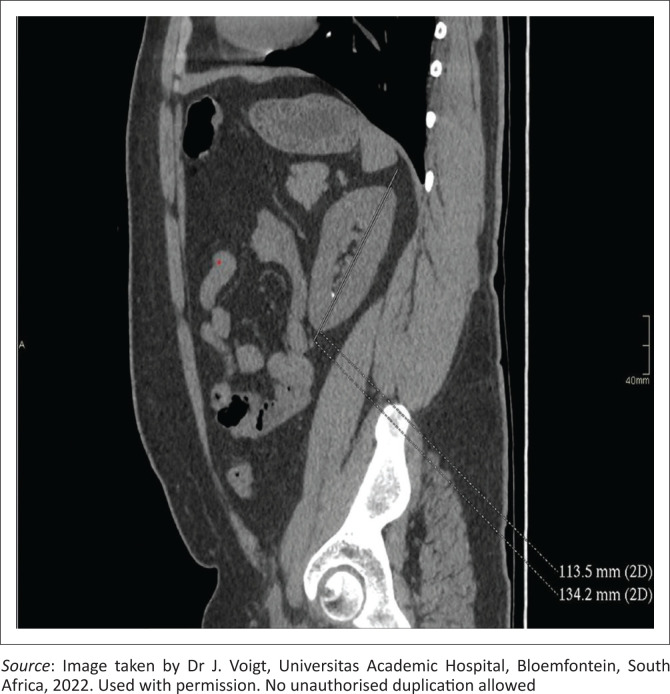
Calculating renal and perirenal fat lengths with manual tracing software at the level of the maximum kidney length in the cranio-caudal plane.

On the sagittal view at the point of the maximum cranio-caudal renal length, the renal and the perirenal fat lengths were measured for both pathological and healthy kidneys (see Figure 2 – example image obtained from one of the study participants).^10^

Since the kidney has a complex ellipsoid shape, the ellipsoid equation (Equation 1) was applied to calculate both renal and PFVs from the measured areas and lengths^14^:

$$Volume=length\times height\times width\times \left(\frac{\pi }{6}\right)$$ [Eqn 1]

The calculated kidney and hilum volumes were then subtracted from the PFV to determine the true amount of fat surrounding the kidney.^10^

The PFT was measured on an axial slice at the level of the renal vein, from the posterior border of the kidney to the inner border of the posterior abdominal wall (see Figure 1 – example image obtained from one of the study participants).^13^ This allowed us to compare the PFT’s of both kidneys, as well as the PFTs and PFVs in the same individual.

### Study design

A retrospective cross-sectional study was done in the radiology department of Universitas Academic Hospital (UAH) in Bloemfontein, South Africa.

A total of 140 patients, comprising 91 males and 49 females, were included. The sample size gives an approximate number of patients diagnosed with kidney stones seen per year at this institution.

The study population included all adult patients, aged between 18 years and 99 years, who were diagnosed with unilateral nephrolithiasis between 2017 and 2023. Both sexes and all racial groups were represented in the study population.

Bilateral nephrolithiasis and or renal lesions that distort the renal cortex were excluded from the study.

### Statistical analysis

Descriptive statistics, namely frequencies and percentages, were used as the summary statistics for categorical data. The distribution of numerical data was skewed; therefore, the median and interquartile range were used as the summary statistics.

The biological sex associations regarding kidney values were calculated for categorical data and described using the chi-square test, and for numerical data, the Kruskal–Wallis test was used. The difference between normal and pathological kidneys was calculated and described using the Signed-Rank test for paired data. The correlation between PFT and PFV was calculated using Spearman’s correlation for non-parametrical data. The analysis for this study was generated using SAS software.^1^ The analysis was done by the Department of Biostatistics, University of the Free State.

### Ethical considerations

Ethical clearance to conduct this study was obtained from the University of Free State and Health Sciences Research Ethics Committee (No. UFS-HSD2022/1886).

## Results

A total of 140 patients were investigated, comprising 49 females and 91 males. Table 1 demonstrates the patients’ characteristics. Left-sided kidney stones were more common in both sexes.

**TABLE 1:** Characteristics of patients (*N* = 140).

Variable	Frequency	%	Age (years)		Interquartile range	Lateralisation of stone	
			Median	Range		Side	%
Female	49	35	49.2	19.2–79.6	34.5–57.4	Left	61.2
Male	91	65	42.3	19.4–80.0	33.4–56.2	Left	60.4

For both sexes, we found that the PFV was larger surrounding the pathological stone-bearing kidney compared to the normal kidney without a kidney stone (Table 2). Females had a median PFV of 118.1 cm^3^ surrounding the pathological kidney compared to 86.6 cm^3^ surrounding the normal kidney.

**TABLE 2:** Perirenal fat volume and perirenal fat thickness results for both normal and pathological kidneys per sex.

Variable	Result (median)	Interquartile range	Min – Max
**Females (*n* = 49)**			
**Pathological kidney**			
PFT (mm)	8.8	6.3–14.0	1.2–59.2
PFV (cm^3^)	118.1	69.9–167.2	21.6–664.4
**Normal kidney**			
PFT (mm)	6.8	3.6–12.2	2.3–38.2
PFV (cm^3^)	86.6	63.3–133.6	20.3–387.6
**L+R kidney**			
Total PFV (cm^3^)	198.2	139.8–301.6	50.3–1052.1
**Males (*n* = 91)**			
**Pathological kidney**			
PFT (mm)	13.1	7.3–20.0	1.8–53.9
PFV (cm^3^)	147.1	79.4–281.0	8.0–704.2
**Normal kidney**			
PFT (mm)	11.9	7.1–19.4	1.3–38.9
PFV (cm^3^)	143.0	64.2–215.2	22.2–1820.9
**L+R kidney**			
Total PFV (cm^3^)	295.8	143.5–490.8	36.8–2216.2

PFV, perirenal fat volume; PFT, perirenal fat thickness; min, minimum; max, maximum.

Males had a median PFV of 147.1 cm^3^ surrounding the pathological kidney compared to 143 cm^3^ surrounding the normal kidney.

Additionally, the PFT was higher posterior to the pathological kidney compared to the normal kidney (Table 2). Females had a median PFT of 8.8 mm posterior to the pathological kidney compared to 6.8 mm posterior to the normal kidney. Males had a median PFT of 13.1 mm posterior to the pathological kidney compared to 11.9 mm posterior to the normal kidney.

The PFV surrounding the stone-bearing kidney was significantly higher than that surrounding the healthy, non-stone-bearing kidney (*p* < 0.0001) (Table 3). Similarly, the PFT was significantly higher posterior to the stone-bearing kidney (*p* < 0.0001).

**TABLE 3:** The difference of perirenal fat volume and perirenal fat thickness between normal and pathological kidneys in all patients.

Variable	Normal kidney			Pathological kidney			*p*-value
	Median	IQR	Min–Max	Median	IQR	Min–Max	
PFV (cm^3^)	114.8	63.8–198.5	20.2–1820.9	135.3	75.5–222.3	8.0–704.2	< 0.0001
PFT (mm)	10.4	4.8–17.3	1.3–38.9	11.5	6.4–18.5	1.2–59.2	< 0.0001

IQR, interquartile range; PFV, perirenal fat volume; PFT, perirenal fat thickness; min, minimum; max, maximum.

**FIGURE 3:**
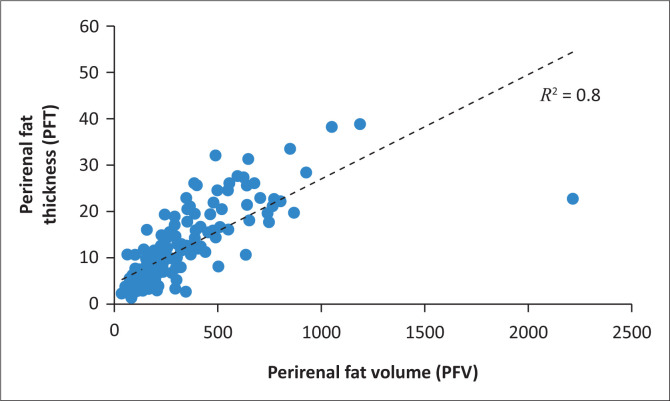
Correlation[AQ1] of perirenal fat thickness and perirenal fat volume.

A significant association between the PFV and the PFT has been found with correlation coefficient *R* = 0.8 (*p* < 0.0001) (Figure 3).

There was a slight trend of smaller PFVs and total PFVs in males compared to females; however, no statistically significant difference between biological sex, PFV and a fat-storing distributing pattern was found (*p* = 0.3749).

Intra-observer variability was assessed using the coefficient of variation (CV). Measurements were repeated three times, and the mean and standard deviation were calculated for each measurement parameter per patient. Variability was low overall, with most CVs below 5%, indicating excellent reproducibility. Median CVs for perirenal fat length were 1.11% (interquartile range [IQR]: 0.73% – 1.71%) on the left and 1.31% (IQR: 0.66% – 2.04%) on the right, while kidney length showed excellent reproducibility with median CVs of 0.86% bilaterally. Kidney area also demonstrated low variability (median CV approximately 2.4%). Perirenal fat thickness showed higher variability (median CV approximately 6% – 7%) but remained within acceptable limits.

## Discussion

Over the last few years, studies have aimed towards explaining the sex predilection of nephrolithiasis.^1,2,15^ The focus is currently shifting from obesity as being a risk factor for nephrolithiasis to sex-based fat-storing distribution patterns as a possible explanation.^13^

A recent French study proved that BMI and anthropometry are not reliable predictors of kidney stone formation.^13^ Body mass index is not able to distinguish between muscle and fat as it is not a calculation of the percentage of fat or the percentage of muscle per individual,^4,13^ and thus cannot be used to investigate the true correlation with nephrolithiasis.^10,13^

The concept of fat distribution patterns has been introduced lately.^10,13^ Males exhibit a visceral fat distribution, where fat accumulates around intra-abdominal organs, such as the kidneys. In contrast, females tend to have a subcutaneous fat distribution, accumulating fat around the central abdomen.^10,13^

Several studies found a positive association between PFV and nephrolithiasis.^4,10,13,16^ The perirenal adipose tissue, a visceral fat component, plays a key role in maintaining cardiovascular and renal homeostasis.^11^ This tissue comprises a unique microenvironment, including white and brown adipocytes, vascular structures, inflammatory cells and a network of nerves and lymphatics.^11^ Perirenal visceral adipose tissue is closely associated with inflammatory and obstructive physiological responses that affect renal blood flow, lymphatic drainage, electrolyte reabsorption, urine excretion and renal interstitial pressure.^4,10,17^ Renal lipomatosis is responsible for renal vein and lymphatic compression at the level of the renal hilum and subsequently causes an increase in renal interstitial pressure, which results in sodium reabsorption.^4,10,17^ Elevated levels of sodium can cause hypertension, which in turn is associated with renal stone disease.^4^ Hypertension has been proven to increase the formation of kidney stones fivefold.^4,18^

One study found a significant increase in PFV in patients presenting with unilateral calcium oxalate kidney stones.^10^ Another study found a direct association between PFV and uric acid levels.^19^ Tastemur et al. performed a case-control study on patients with unilateral nephrolithiasis as the case group and healthy patients without kidney stones as the control group. They found that the PFV surrounding the stone-bearing kidney was higher than the non-stone-bearing kidney in the same individual.^4^ They also determined that when combining the PFV of both kidneys in the same individual, a bilateral combined PFV of > 387 cm^3^ in the same individual could be regarded as an independent risk factor for kidney stone formation as the combined PFV of the case group was much larger than in the control group.^4^

If males contain larger PFV compared to females, and if perirenal fat is associated with kidney stone formation, this might explain the male predominance of nephrolithiasis.^13^

Perirenal fat volume can be measured using US, CT or MRI.^12^ Each modality offers varying levels of accuracy, precision, time efficiency, reproducibility and radiation exposure, depending on the specific clinical question being addressed.^12^ Ultrasound is cost-effective but operator-dependent. Computed tomography poses a high radiation risk; however, more accurate 3D volumetric or manual tracing software can be applied to calculate organ volumes.^12,13^ Magnetic resonance imaging is expensive and sometimes only available in tertiary referral centres in the public sector and private sector healthcare facilities.

One study compared the different methods for calculating kidney volume.^12^ The kidney’s ellipsoid shape makes calculations based on simple formulas difficult and inaccurate.^12,14^ The ellipsoid method, performed on CT, was chosen for this study because no 3D calculating software was available. Although the ellipsoid method may be less reproducible and precise than other available methods, it still provides a good estimate and understanding of renal and perirenal volumes while being less time-consuming.^12,14^ For the purpose of this study, the ellipsoid method provided a good understanding of the calculated volumes.

Our study demonstrated a statistically significant association between PFV and nephrolithiasis, postulating a clear causative link. The PFV surrounding the stone-bearing kidney was larger than that PFV surrounding the non-stone-bearing kidney in the same individual. Our results correlated with previous studies that larger PFVs are related to kidney stone formation in the studied population.

A recent study concluded that the time-efficient, simple measurement of PFT on CT at the level of the renal vein could be used as a reliable, quick estimate of PFV since a statistically significant correlation between these two variables had been found.^13^ Another study confirmed that PFT could function as a risk stratification of renal stone recurrence since they proved a significant association between PFT and unilateral nephrolithiasis.^9^ We also found a strong association between PFV and PFT. Perirenal fat thickness can possibly be used as a simple, time-efficient substitute for calculating PFV and possibly predict the formation of kidney stones, as previous studies found.^9,13^

Unexpectedly, we did not observe a statistically significant difference in PFV between males and females. Therefore, we cannot conclude that a relationship exists between PFV, sex and nephrolithiasis in the studied South African population.

To the best of our knowledge, no similar study has been conducted on the South African population or elsewhere on the African continent.

We included a diverse, multicultural cohort of individuals of all ages and sexes from Central South Africa. To eliminate inter-observer bias, all calculations were performed by a single radiology registrar. Measurements were repeated three times to calculate averages and minimise intra-observer bias, making the process time-intensive. Additionally, we excluded patients with deforming cortical lesions that could potentially influence PFV.

A limitation of this study, attributable to its retrospective nature, is the lack of consideration for and inclusion of factors such as BMI, dietary habits, comorbidities, medication, stone composition and urine analysis. Additionally, as images were evaluated retrospectively, the imaging acquisition protocols were not standardised, which limited the establishment of formal quantitative imaging biomarkers. Intra-observer variability was low overall, with median CV values below 5% for kidney area, kidney length and perirenal fat length, indicating good reproducibility. Perirenal fat thickness demonstrated slightly higher variability (median CV approximately 6% – 7%) but remained within acceptable limits. This likely reflects the smaller absolute values of PFT measurements, where minor measurement differences will translate into greater relative variability.

International research postulates that the visceral fat-storing pattern demonstrated in men renders higher PFV values in male patients compared to female patients. However, no statistically significant sex-based differences were identified in our study population. Future research focussed on other sub-Saharan African populations should evaluate this.

A larger sample size, along with verification studies conducted in other regions of the country or continent, would be beneficial for comparing results. Further exploration of the role of perirenal fat would enhance our understanding of the pathophysiology of kidney stones and inform strategies for their prevention.

## Conclusion

The PFV surrounding a stone-bearing kidney was found to be significantly higher than that surrounding a non-stone-bearing kidney in the same individual.

An association between PFT and PFV was demonstrated. Although further verification studies are required, PFT could potentially serve as an additional, briefer measurement to relate PFV and PFT to stone formation.

In the South African population studied, no association was observed between biological sex and PFV. Future studies with larger sample sizes in South Africa would provide further insights into this topic.

## Footnotes

1 Copyright, SAS Institute Inc. SAS and all other SAS Institute Inc. product or service names are registered trademarks or trademarks of SAS Institute Inc., Cary, NC, USA.

## References

[1] Trinchieri A, Montanari E. Prevalence of renal uric acid stones in the adult. Urolithiasis. 2017;45(6):553–562. 10.1007/s00240-017-0962-5 28258472

[2] Scales CD, Smith AC, Hanley JM, Saigal CS, Urologic Diseases in America Project. Prevalence of kidney stones in the United States. Eur Urol. 2012;62(1):160–165. 10.1016/j.eururo.2012.03.052 22498635 PMC3362665

[3] Meyers AM, Naicker S. Nephrolithiasis (part 1): Epidemiology, causes and pathogenesis of recurrent nephrolithiasis. S Afr Med J. 2021;111(10):930–933. 10.7196/SAMJ.2021.v111i10.15988

[4] Tastemur S, Senel S, Olcucuoglu E, Uzun E. Evaluation of the relationship between fat volume and nephrolithiasis. Curr Med Imaging Rev. 2021;18(4):398–403. 10.2174/1573405617666211130154127 34847847

[5] Carbone A, Al Salhi Y, Tasca A, et al. Obesity and kidney stone disease: A systematic review. Minerva Urol Nefro. 2018;70:393–400. 10.23736/S0393-2249.18.03113-2 29856171

[6] Taylor EN, Stampfer MJ, Curhan GC. Obesity, weight gain, and the risk of kidney stones [homepage on the Internet]. 2005 [cited 2022 Oct 05]. Available from: http://jama.jamanetwork.com/10.1001/jama.293.4.455 15671430

[7] Sarica K. Obesity and stones. Curr Opin Urol. 2019;29:27–32. 10.1097/MOU.0000000000000557 30308572

[8] Ekeruo WO, Tan YH, Young MD, et al. Metabolic risk factors and the impact of medical therapy on the management of nephrolithiasis in obese patients. J Urol. 2004;172(1):159–163. 10.1097/01.ju.0000128574.50588.97 15201761

[9] Huang H, Chen S, Zhang W, et al. High perirenal fat thickness predicts a greater risk of recurrence in Chinese patients with unilateral nephrolithiasis. Ren Fail. 2023;45(1):2158870. 10.1080/0886022X.2022.2158870 36637005 PMC9848376

[10] Lama DJ, Safiullah S, Yang A, Okhunov Z, Landman J, Clayman RV. Three-dimensional evaluation of perirenal fat volume in patients with nephrolithiasis. Urolithiasis. 2018;46(6):535–541. 10.1007/s00240-018-1047-9 29500620 PMC6196721

[11] Grigoraș A, Balan RA, Căruntu ID, et al. Perirenal adipose tissue – Current knowledge and future opportunities. J Clin Med. 2021;10(6):1291. 10.3390/jcm10061291 33800984 PMC8004049

[12] Sharma K, Caroli A, Van Quach L, et al. Kidney volume measurement methods for clinical studies on autosomal dominant polycystic kidney disease. PLoS One. 2017;12(5):e0178488. 10.1371/journal.pone.0178488 28558028 PMC5448775

[13] Favre G, Grangeon-Chapon C, Raffaelli C, François-Chalmin F, Iannelli A, Esnault V. Perirenal fat thickness measured with computed tomography is a reliable estimate of perirenal fat mass. PLoS One. 2017;12(4):e0175561. 10.1371/journal.pone.0175561 28423008 PMC5396915

[14] Zakhari N, Blew B, Shabana W. Simplified method to measure renal volume: The best correction factor for the ellipsoid formula volume calculation in pretransplant computed tomographic live donor. Urology. 2014;83(6):1444.e15–1444.e19. 10.1016/j.urology.2014.03.005 24862398

[15] Jones J, Kearns C, Shah V. Urolithiasis [homepage on the Internet]. 2009 [cited 2023 Jul 15]. Available from: Radiopaedia.org

[16] Uyeturk U, Dagıstan E, Aktas G, Ozyalvacli ME, Tekce H, Yilmaz B. National journal of medical research association between urinary stone disease and perirenal tissue thickness [homepage on the Internet]. Vol. 4. 2014 [cited 2022 Oct 05]. Available from: http://www.textcheck.com/certificate/B6dqFF

[17] Montani JP, Carroll JF, Dwyer TM, Antic V, Yang Z, Dulloo AG. Ectopic fat storage in heart, blood vessels and kidneys in the pathogenesis of cardiovascular diseases. Int J Obes Relat Metab Disord. 2004;28(Suppl 4):S58–S65. 10.1038/sj.ijo.0802858 15592488

[18] Curhan GC, Willett WC, Rimm EB, Speizer FE, Stampfer MJ. Body size and risk of kidney stones. J Am Soc Nephrol. 1998;9(9):1645–1652. 10.1681/ASN.V991645 9727373

[19] Jiang M, Li M, Liu C, et al. Perirenal fat volume is positively associated with serum uric acid levels in Chinese adults. Front Endocrinol (Lausanne). 2022;13:865009. 10.3389/fendo.2022.865009 35600604 PMC9120634

